# Feeding by slugs on maize imposes variable productivity costs but can induce compensatory growth under some conditions

**DOI:** 10.1002/ps.70148

**Published:** 2025-08-22

**Authors:** John F. Tooker, Matthew T. Boucher, John M. Wallace, Margaret R. Douglas

**Affiliations:** ^1^ Department of Entomology The Pennsylvania State University University Park PA USA; ^2^ Department of Plant Science The Pennsylvania State University University Park PA USA; ^3^ Department of Environmental Studies & Environmental Science Dickinson College Carlisle PA USA

**Keywords:** *Deroceras reticulatum*, herbivory, mollusk, no‐till farming

## Abstract

**BACKGROUND:**

In contrast to herbivorous insect pests, relatively little attention has been given to how terrestrial mollusks affect plant productivity. This lack of attention is problematic because in some crop‐growing areas, particularly those that have adopted no‐till farming practices and get ample rain, slugs are often the most challenging invertebrate pests that farmers face, but there is a poor understanding of how slug feeding on young crop plants influences their growth. To address this knowledge gap, we conducted a greenhouse experiment that exposed two different ages of young maize plants to slug and mechanical damage and then tracked their growth and development for 6 weeks. To complement our greenhouse experiment, we also analyzed legacy data of slug damage and maize productivity from three field experiments conducted over six growing seasons in no‐till crop fields in central Pennsylvania, USA.

**RESULTS:**

Results from our greenhouse experiment suggested that slug and mechanical damage are largely similar but also indicated that low levels of slug damage can induce compensatory growth. Notably, some of our field research corroborated that low levels of slug feeding can positively influence maize yield, whereas other results indicated that heavier levels of slug damage can have the expected negative influence on maize yield.

**CONCLUSION:**

Our data suggest that slug damage is similar to mechanical damage and that the influence of slug feeding on maize development or productivity depends on the amount of damage received and the local growing conditions. © 2025 The Author(s). *Pest Management Science* published by John Wiley & Sons Ltd on behalf of Society of Chemical Industry.

## Graphical abstract

Slugs can challenge crop plants grown in no‐till fields. Our results confirm that higher levels of slug feeding can be detrimental to maize plants, but low levels of herbivory by slugs can unexpectedly increase plant growth and occasionally yield.

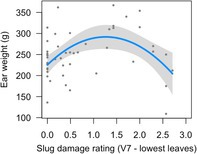

## 1 INTRODUCTION

In natural and agricultural systems, a diversity of herbivores attack plants. These attacks, most of which are committed by insects and mites (Arthropoda), can have clear costs for plant productivity, with seemingly small amounts of arthropod damage decreasing plant reproductive output, fitness, and/or yield.^1^, ^2^ In some cases, however, herbivory can be innocuous for individual plants or result in compensatory growth that can overcome negative influences of tissue removal.^3^, ^4^ Greenhouse and laboratory experiments, often complemented with molecular or biochemical analyses, have revealed that damage to plants by insects goes beyond mechanical damage (*i.e*., wounding), indicating that factors associated with insects (*e.g*., insect saliva) help plants to recognize herbivory and mount appropriate defensive responses.^5^, ^6^, ^7^, ^8^

In many regions, however, insects are not the only pests attacking plants and may not even be the most significant invertebrate pests that crops face. Indeed, in regions that tend to have cool, wet conditions when crops are establishing, slugs (Mollusca: Agriolimacidae, Arionidae) can be among the most challenging pests to manage.^9^, ^10^, ^11^ In the Mid‐Atlantic United States, for example, slugs are significant pests of no‐till field crops, feeding in spring on newly emerging plants, like maize and soybeans, or in autumn on establishing crops like small grains or alfalfa.^10^, ^12^, ^13^, ^14^ The lack of disturbance in no‐till cropping systems provides slugs with stable habitats and ample food that allows their populations to grow year after year.^15^ Further, because fields are not tilled, residue from preceding crops remains on the soil surface, where it retains moisture and provides slugs places to hide.^16^ Lastly, pesticide use in these crops can disrupt natural‐enemy communities, including insect predators and parasitic nematodes, potentially releasing slug populations from biotic control and increased crop damage.^17^, ^18^ Because slugs are regional pests, their interactions with crop plants have not received as much research attention as interactions of plants with herbivorous insects.

Nevertheless, previous research has established that herbivory by slugs can influence plant communities and plant performance. For example, studies of grassland systems suggest that slugs can mediate plant community composition due to differences in herbivory among species.^19^ Moreover, feeding preferences of slug species reflect their sensitivity to plant secondary metabolites and slug feeding on plants appears to have selected for higher concentrations of secondary metabolites in some plant species.^20^, ^21^ Like insects, feeding by slugs and snails can also trigger inducible plant defenses that may be mediated by specific cues present in their mucus and other secretions.^22^, ^23^

Taken together, these previous studies suggest that slug feeding can impose costs on some plant species, but relative to mechanical damage it is unclear how costly slug feeding is for crop plants and whether they can recover from early season slug damage. Mechanical damage to young corn plants (six‐leaf stage or younger) has mostly limited growth and productivity, but some hybrids have shown yield increases.^24^, ^25^, ^26^, ^27^ More recent research found that corn plants completely defoliated at the three‐leaf stage had similar grain yield to undamaged plants, suggesting that corn plants can recover from considerable mechanical damage.^28^, ^29^ Therefore, it seems that mechanical damage to corn plants largely hampers plant productivity but is context dependent in its effects. We are unaware, however, of previous research comparing the influence of slug feeding and mechanical wounding on maize growth; such research could reveal costs of slug feeding that may be associated with slug‐specific cues from their mucus or saliva. Furthermore, some research used to develop recommendations for farmers has used only mechanical damage,^30^, ^31^ which may not represent fully the influence that slugs have on plants.

To fill these knowledge gaps, in this paper we evaluated the influence of slug feeding and mechanical wounding on young, greenhouse‐grown maize plants and assessed maize productivity for 6 weeks after slug and mechanical damage. We hypothesized that slug feeding, and mechanical wounding would both negatively influence plant growth, but that slug feeding would be more damaging because slug‐specific cues would trigger costly plant defense responses, leading to greater reductions in plant growth. To complement our greenhouse‐based research, we also explored influences of early season slug damage on maize productivity using data from three field experiments conducted between 2010 and 2015. The first of these experiments assessed the effect of early season slug damage on maize productivity. The second and third experiments originally explored the influence of crop rotations^32^ or neonicotinoid seed treatments,^33^ respectively, on slugs, their predators, and crop yield, but also included assessments of slug damage that can provide insight on the influence of slug feeding on plant growth and productivity. Like our greenhouse work, in our field research we expected that heavier levels of slug feeding would limit maize growth and productivity.

## 2 MATERIALS AND METHODS

### 2.1 Greenhouse‐based experiment

*Slug colony*: In the Mid‐Atlantic U.S., grey garden slug (*Deroceras reticulatum*) is typically the most common and damaging slug species in large acreage no‐till crop fields.^10^ To study the influence of this species on corn in a greenhouse setting, we collected about 600 *D. reticulatum*, from underneath 0.09 m^2^ pieces of white roofing shingles (color: Shasta White; Owens Corning, Toledo, OH) placed along the edge of a mown lawn and long‐term pasture behind Merkle Laboratory in University Park, PA 16802 (40°49′10.2″N, 77°51′33.8″W). Slugs were collected from under traps daily in the morning from April 2021 – May 2021, and again from late August 2021 – October 2021. Slugs were transferred into plastic containers (23 cm L × 17 cm W × 8.5 cm H, 2130 mL volume; Ziploc® brand, SC Johnson & Son, Inc; Racine, WI) with moist potting mix (Pro‐Mix Bx Mycorrhizae; Premier Horticulture Inc; Quakertown, PA) spread evenly across bottoms of containers to a height of 2.54 cm. To prevent escape, we securely sealed the accompanying lids that had been modified for ventilation by cutting rectangular holes (14 × 7‐cm) over which we glued fine mesh. For food, we provided slugs with a Petri dish containing 2–3 bok choy leaves, 4–6 baby carrots, and 1–3 napa cabbage leaves purchased from a local grocery store. We added wet paper towel (~38 cm^2^) to containers to maintain moisture.

In each container, we maintained roughly 100 slugs and replaced food and paper towels weekly. When replacing resources, we examined food, paper towels, and Petri dishes for slugs and eggs, removing eggs and juvenile slugs with a paint brush and adult slugs with forceps and returning all to the container. We removed dead slugs and fungal growth from soil when we replaced food. We kept containers in a low‐temperature incubator (Model 2015; VWR International, Radnor, PA) without lights (0:24 D:N cycle) at 10–15°C. The incubator had no humidity controls, so to increase humidity we added several large containers of water to the bottom shelf. Monthly, we transferred slugs to new containers with fresh soil and transferred old soil to separate, clean containers. This allowed us to clean the old containers and to separate soil containing eggs from adults so that we could maintain individual cohorts.

For our greenhouse experiment, we ran all replicates using the original, wild‐caught cohort of adult slugs collected from behind Merkle Lab. Slugs were not re‐used in this experiment. We used adults in our experiment because we needed fewer of them to populate our cages and their damage is more pronounced, so it can be more confidently quantified.

*Experimental design*: To evaluate the influence of slug feeding and mechanical wounding (*i.e*., damage type) on young, maize plants, we used a single factor experimental design with five treatments that was conducted with two different starting conditions: damage at corn growth stage V1 or V3. Starting conditions were not considered a factor in the experimental design because damage to V1 or V3 plants was not imposed at the same time.

We planted maize seeds (97D Agrisure Viptera® 3220; Local Seed Co, Memphis, TN) treated with 0.0002 mL Maxim® Quattro fungicide (Fludioxonil, Mefenoxam, Azoxystrobin, Thiabendaz; Syngenta AG, Basel, Switzerland) per seed in plastic pots (16.5 x 12 cm) with moist potting soil (same as above). We planted one seed per pot, roughly one‐inch deep, and added 4 g of Miracle‐Gro® Shake ‘n Feed all‐purpose fertilizer (Miracle‐Gro Lawn Products, Inc; Marysville, OH). We maintained plants in a partially climate‐controlled greenhouse with a 14:10 D:N cycle with lights on from 06:00 to 20:00 at ~25 °C. We watered plants weekly or as needed throughout the experiment. We allowed 125 plants to reach the V1 growth stage before exposing them to damage, and 125 other plants to reach V3 before exposing them damage, for a total of 250 experimental plants. We blocked replicates by time (five blocks for each growth stage) by planting 50 plants at a time (starting in mid‐August and finishing in late November, 2021), with half within a block designated for damage exposure at V1 and the other half at V3.

We exposed five plants per block to one of five damage types (*i.e*., treatments): (1) control; (2) low mechanical (LM); (3) high mechanical (HM); (4) low slug (LS); (5) high slug (HS). To contain slugs on plants and prevent escape into soil, we placed a 5.5‐cm diameter plastic disk cut from transparency film for laser printers (APOLLO VCG7060E; ACCO Brands; Booneville, MS) around the bases of plants, and wrapped moist cotton around stalks, covering the plastic disks. Then, we slid a 45.5 cm tall, 5.5 cm diameter PVC pipe over the plant, pressing slightly into the moist cotton. We secured the pipe by pressing four bamboo stakes into the soil and taping them to the pipe. We wrapped aluminum foil around the top opening of the pipe and secured it with a rubber band to seal the tubes shut. For replicates exposed to slugs, slugs were placed on the plant by dropping them into the open top of the pipe before sealing. For low‐intensity slug damage, we added five slugs to each replicate whereas we added 10 slugs to each replicate for high‐intensity slug damage. Economic thresholds for slugs tend to be based on percent defoliation^34^ and there is a poor understanding of the relationship between numbers of slugs and the amount of damage that they cause, so we figured that twice as many slugs would cause more damage to corn plants. We measured the total mass of slugs added to individual plants prior to releasing slugs onto plants. We treated plants designated for mechanical damage or controls the same, so they too received PVC arenas but had no slugs added. This experimental design excluded light from plants, and the slugs we put on them, for 48 h.

After 48 h, we removed PVC arenas, moist cotton, plastic disks, and slugs. Next, to create low and high mechanical damage for designated replicates, we visually assessed amounts of damage inflicted by slugs on each plant and replicated it using a 0.3‐cm hole punch to remove approximately that same amount of tissue. We aimed to cause mechanical damage in similar positions on leaves as that imposed by slugs. For each slug‐damaged plant, we created one plant in the mechanically damaged treatment, but we did not keep track of these pairs and did not conduct paired statistical analyses with them. Due to our experimental design, slug damage began 48 h prior to the mechanical damage.

To quantify amounts of tissue that slugs or mechanical damage removed, we measured amounts of missing leaf area from one leaf of each replicate; for V1 replicates we measured the V1 leaf, and for V3 replicates we measured the V3 leaf. We did not harvest leaves but flattened the one leaf per plant on a white clipboard with a piece of transparency film (for laser printers) and then took a picture of each with a smartphone. Each image included a yellow 7.5 × 7.5‐cm sticky note to provide a scale. We then calculated the final leaf area using ImageJ.^35^ After we removed the PVC arenas, we measured plant height (measured from soil to the extended tip of the youngest leaf with a full collar) and stalk diameter (at the soil) for each plant and kept them in the greenhouse for 6 weeks after damage exposure. We then measured plant height and stalk diameter each week. After the sixth week, plants were cut at the soil surface and placed into individual brown paper bags and dried for 2 weeks in the greenhouse. After drying, we weighed aboveground dry biomass of every plant.

### 2.2 Field‐based studies

To characterize the relationship between slug damage and corn yield under field conditions, we re‐analyzed data from three previous field experiments in central Pennsylvania, USA (Penn State's Russell E. Larson Agricultural Research Station; Pennsylvania Furnace, PA; 40.721384, −77.919952). These experiments investigated the influence of (i) slug damage early in the growing season on maize productivity,^40^ (ii) diverse crop rotations on maize and its invertebrate community,^32^ (iii) neonicotinoid seed treatments on slugs, their predators, and crop yield.^33^

The first two experiments were part of a dairy cropping systems project,^32^ and our analyses here use data collected in the ‘control’ rotation, a no‐till maize‐soy rotation that was managed using practices typical of the region. The study was a randomized block design (*n* = 4 plots, each 27.4 × 36.6 m), with split‐plots (*n* = 8, each 27.4 × 18.3 m) that differed in fertility management (chemical fertilizer *vs*. dairy manure). Each split‐plot was further divided into two split‐split plots (*n* = 16, 27.4 × 9.1 m) to facilitate sampling and application of additional experimental treatments.^32^ In 2010, before experimental treatments were fully realized, we followed individual maize plants over one growing season to better understand the influence of early‐season slug damage on grain yield. We focused our sampling in each split‐plot that received broadcast applications of manure (details available in Douglas 2012; Busch *et al*., 2020).^32^, ^40^ In each split‐split‐plot (*n* = 8), we selected twelve maize plants in early July (2 July 2010) in a stratified random pattern and marked them with flagging tape around their bases. All maize plants were within the center eight rows of each split‐split‐plot to avoid edge effects and were at growth stage V7. Seven independent observers rated each maize plant for slug damage. Because maize plants were beyond the stage where slug damage is typically most severe, we rated only the bottom four leaves to capture damage to an earlier growth stage of the plant. Those leaves were rated on a damage scale as follows: 0 = no damage; 0.4 = <10% defoliation, 1 = 10–25% defoliation, 2 = 25–50% defoliation, 3 = 50–75% defoliation, and 4 = 75–100% defoliation. In October, when maize plants had dried down and were ready for grain harvest (23 October 2010), we collected the marked maize plants and weighed their whole ears; we assumed that ear mass strongly correlated with grain yield.

In the same cropping systems experiment described above, we made a larger effort to understand the influence of slugs on crop growth by using 5 years of data (2011–2015). The variables used in the analysis were grain yield and the percent of maize seedlings at stage V5 that were damaged by slugs. Because caterpillar damage may have affected yield independently of slugs, we also measured percent of seedlings damaged by cutworms or other caterpillars at stage V5. For full details of the methods see Busch *et al*. (2020).^32^

### 2.3 Statistical analyses

#### 2.3.1 Greenhouse‐based experiment

We conducted all analyses in R^36^ and analyzed V1 and V3 replicates separately. Figures were generated in R using the ggplot2 library.^37^ Using the glmmTMB library in R, we analyzed the effects of slug and mechanical damage on maize height and stalk diameter over time with a repeated measures generalized linear mixed model (GLMM).^38^ We fit the model to a Gaussian distribution, including treatment, week, and their interaction as fixed effects and sample nested within week as random effects to account for repeated measures. We used the emmeans library to make pairwise comparisons of estimated marginal means between treatments within individual weeks to determine how damage to young plants affects growth over time using the Tukey method for multiple comparisons.^39^ We analyzed the effect of damage type on final plant biomass using a GLMM, including treatment as a fixed effect and block start date as a random effect. We fit the model to a Gaussian distribution and made post‐hoc comparisons as described above. We analyzed the effect of damage type on leaf area in the same way. We used linear regression in base R to assess how leaf area related to final plant height, final stalk diameter, and biomass. Using only replicates that were treated with slugs, we used linear regression to assess how the total slug mass placed on plants related to leaf area, biomass, final height, and final stalk diameter.

#### 2.3.2 Field‐based experiments

To analyze the relationship between slug damage and maize yield for 5 years of data from 2011 to 2015, we used a mixed modeling approach in R^36^ with the ‘lmer’ function, package ‘lme4’.^41^ Maize yield was the response variable, and we fit a model for the percent of plants damaged by slugs. Covariates included caterpillar damage and fertility treatment. Random effects of year, main plot, and split‐split‐plot nested in main plot were included to account for non‐independence over space and time. The model fit was checked as suggested by Zuur *et al*. (2007),^42^ including the variance inflation factor for multicollinearity among predictors (values >5 considered concerning). Significance of terms was tested using t‐tests with Kenward‐Roger degrees of freedom as calculated by package ‘lmerTest’.^43^

While the cropping systems experiment included a range of plots and years with variable slug pressure, it did not include heavy slug pressure. To gain insight on the effects of slugs under a wider range of conditions, we also analyzed data from one year (2012) of a seed‐treatment experiment that included plots with larger slug populations (two to five slugs per shingle trap) and heavy slug damage to corn plants (many assessments found 100% of plants damaged by slugs).^33^ In 2012, the study was a Latin‐square design in which the treatment of interest was presence or absence of a neonicotinoid seed treatment (n = 12 plots, six per treatment, each 30.5 × 32 m), at a site that had been under no‐till management for at least 7 years. The main variables in the analysis were slug damage (4‐point scale as described above) to maize seedlings at V3 and grain yield. We also included percent of seedlings damaged by cutworms at V3; this was the only other significant early‐season pest damage we observed. For full details of the methods see Douglas 2016.^33^

Because our experiments in 2010 and 2012 each only used one year of data, we were able to use a simpler linear modeling approach to investigate the relationship between slug damage and maize yield. To examine for 2010, whether early season slug damage related to eventual grain yield of individual plants, we fit a quadratic regression (function ‘lm’) with ear weight as the response variable and slug damage at V7 and its square as predictors. We chose to fit a quadratic model because initial inspection of the data showed a non‐linear relationship. For 2012, similar to results in dry years from a previous study (Byers and Calvin, 1994), we fit a linear regression (function ‘lm’) with yield as the response variable and slug damage at V3 as the main predictor variable. Seed treatment and cutworm damage were included as covariates.

In all analyses for our field experiments, effects were considered significant at *P* < 0.05 and marginally significant at *P* < 0.10, and we present means with standard errors (X ± SE) unless otherwise noted.

## 3 RESULTS

### 3.1 Greenhouse‐based experiment

**Figure 1:**
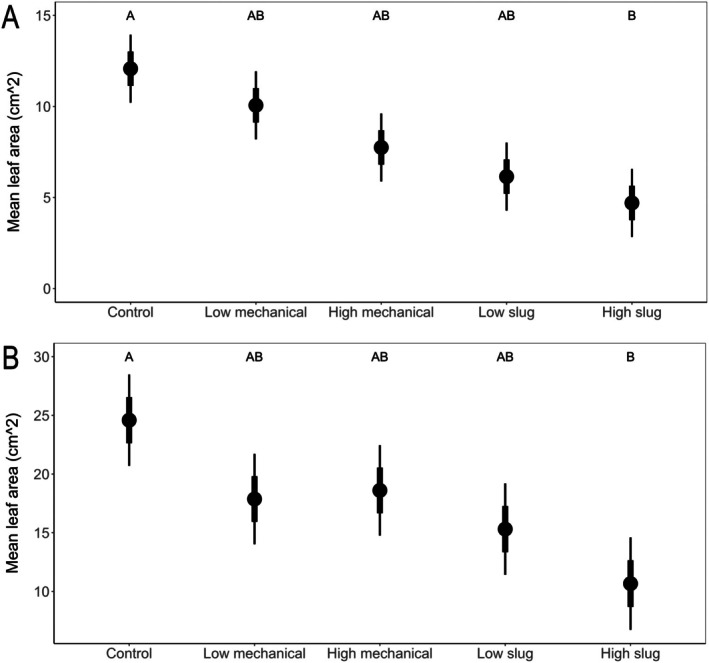
Estimated marginal mean leaf area of plants that experience five damage types at (A) V1 and (B) V3. Black points represent the mean, thick bars represent mean ± SE, and thin bars represent mean ± 95% CI. Treatments are not significantly different at *P* < 0.05 if they share a letter.

*Establishment of treatments*: When assessed by the areas of the V1 leaves remaining after damage, plants with HM damage had lower leaf area (*i.e*., more plant tissue removed) than plants with LM damage (*t*‐ratio = 3.10, *P* = 0.021), but HS and LS damage were similar (*t*‐ratio = 1.91, *P* = 0.32; Fig. 1(a)). For leaf area remaining of V3 leaves, the treatments LM, HM, and LS, received similar levels of injury (*e.g*., LM *vs*. LS: *t*‐ratio = 1.27, *P* = 0.71) whereas the HS treatment removed more leaf area than either mechanical treatment (Fig. 1(b); LM *vs*. HS: *t*‐ratio = 3.53, *P* = 0.005). Overall, for both growth stages, the amounts of tissue removed by slugs on the V1 or V3 leaf tended to be greater than that removed by mechanical damage; as a result, slug‐damaged plants tended to have lower remaining leaf area (Fig. 1). Unfortunately, based on amounts of damage to one leaf per plant, we did not establish equivalent amounts of damage on our low and high treatments, but we succeeded in establishing a gradient in damage that was still useful for providing insight on the effects of slug damage on plants.

**Figure 2:**
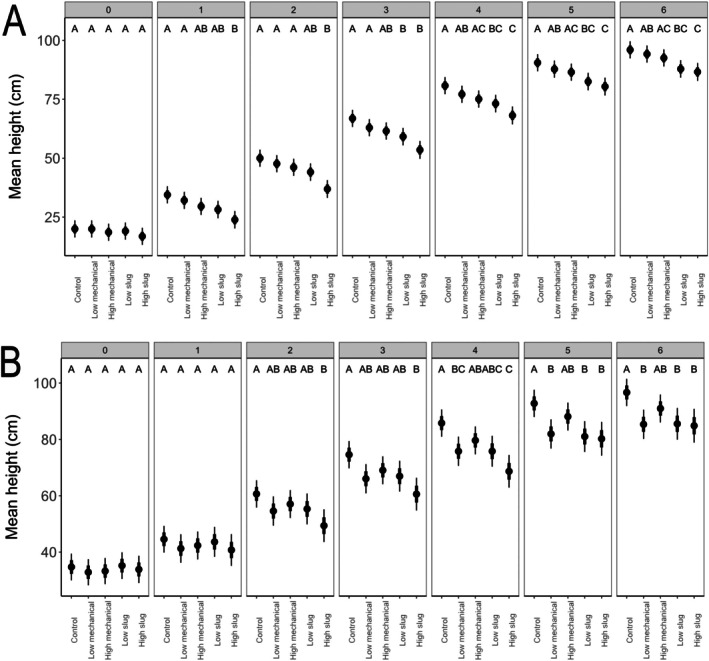
Estimated marginal mean height of plants damaged at (A) V1 and (B) V3 split by week and damage type. Black points represent the mean, thick bars represent mean ± SE, and thin bars represent mean ± 95% CI. Treatments are not significantly different within a week at *P* < 0.05 if they share a letter.

**Figure 3:**
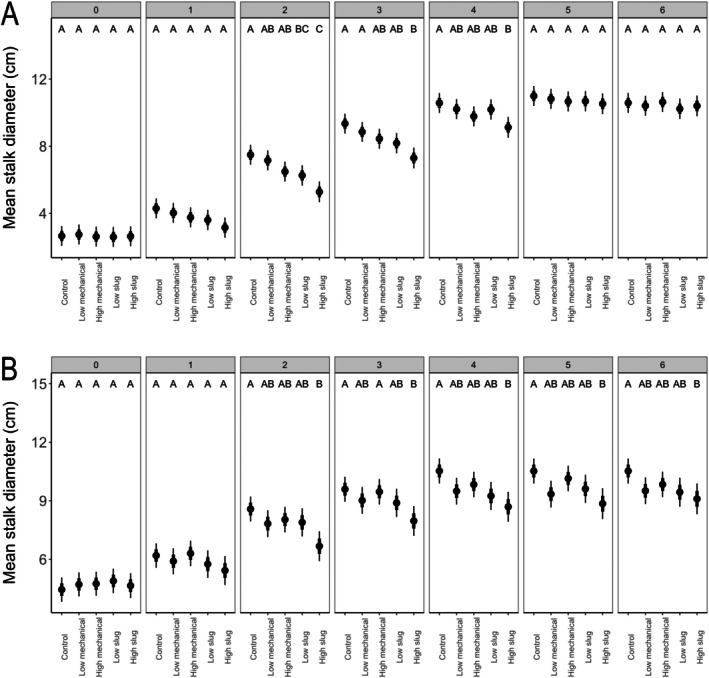
Estimated marginal mean stalk diameter of plants damaged at (A) V1 and (B) V3 split by week and damage type. Black points represent the mean, thick bars represent mean ± SE, and thin bars represent mean ± 95% CI. Treatments are not significantly different within a week at *P* < 0.05 if they share a letter.

*Effects of damage on plant height and stalk diameter over time*: When exposed to damage at V1, differences in plant height emerged gradually (first evident at after week 1; *e.g*., LM *vs*. HS: *t*‐ratio = 3.12, *P* = 0.016) and were most evident after week 6 (Fig. 2(a)). After 6 weeks of growth, plants exposed at V1 to HS damage were significantly shorter than control plants (*t*‐ratio = 3.52.02, *P* = 0.004) and LM plants (*t*‐ratio = 2.85, *P* = 0.037), whereas LS plants were significantly shorter than control plants (*t*‐ratio = 3.07, *P* = 0.019), but similar in height to plants with HM (*t*‐ratio = 1.77, *P* = 0.39) and LM damage (*t*‐ratio = 2.40, *P* = 0.11; Fig. 2(a)). Differences in stalk diameter emerged among treatments after damage (*e.g*., week 2: *e.g*., Control *vs*. LS: *t*‐ratio = 2.86, *P* = 0.035) but dissipated by week six (Fig. 3(a); *e.g*., Control *vs*. HS: *t*‐ratio = 0.41, *P* = 0.99).

When exposed to damage at V3, differences among treatments emerged after week 2. The main consistent result was that HS plant were significantly shorter than control plants from week 2 through week 6 (Fig. 3(b); *e.g*. week 2: Control *vs*. HS: *t*‐ratio = 2.94, *P* = 0.028). After 6 weeks, LS and HS plants were significantly shorter than control plants (LS: *t*‐ratio = 2.95, *P* < 0.027; HS: *t*‐ratio = 3.02, *P* = 0.022), but similar in height to mechanically damaged plants (*P* > 0.05) even though slug damage tended to remove more tissue (Fig. 1(b)). For stalk diameter, plants damaged at V3 had some significant differences among treatments in weeks 2–4 (*e.g*., week 2: Control *vs*. HS: *t*‐ratios = 3.76, *P* = 0.002), but after 6 weeks of growth HS plants had significantly smaller stem diameters than control plants (Fig. 3(b); *t*‐ratio = 2.76, *P* = 0.046).

**Figure 4:**
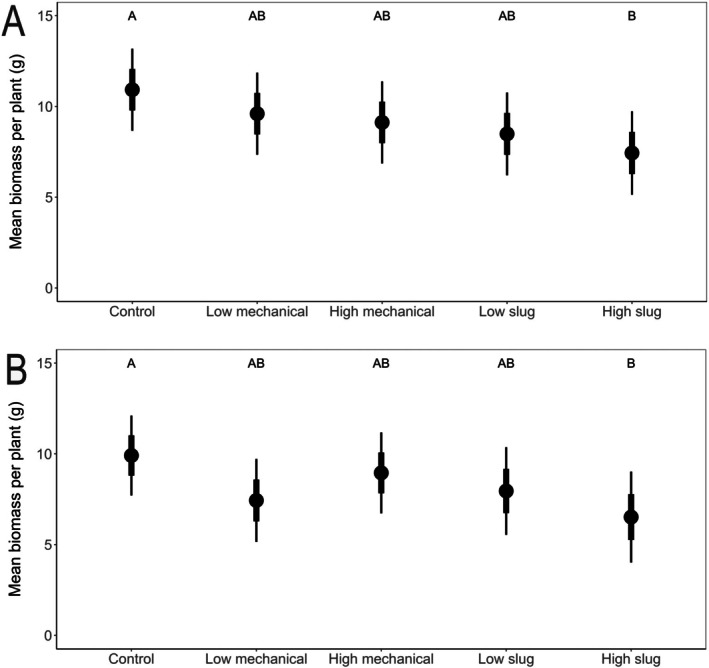
Estimated marginal mean biomass of plants damaged at (A) V1 and (B) V3 split by damage type. Black points represent the mean, thick bars represent mean ± SE, and thin bars represent mean ± 95% CI. Treatments are not significantly different within a week at *P* < 0.05 if they share a letter.

*Effect of damage type on biomass*: When exposed to damage at V1 or V3, HS plants had significantly lower biomass than control plants (V1: *t*‐ratio = 3.46, *P* < 0.05; V3: *t*‐ratio = 2.82, *P* < 0.05), but there were no differences among the other treatments (Fig. 4; *t*‐ratios <2.0, *P* > 0.05).

**Figure 5:**
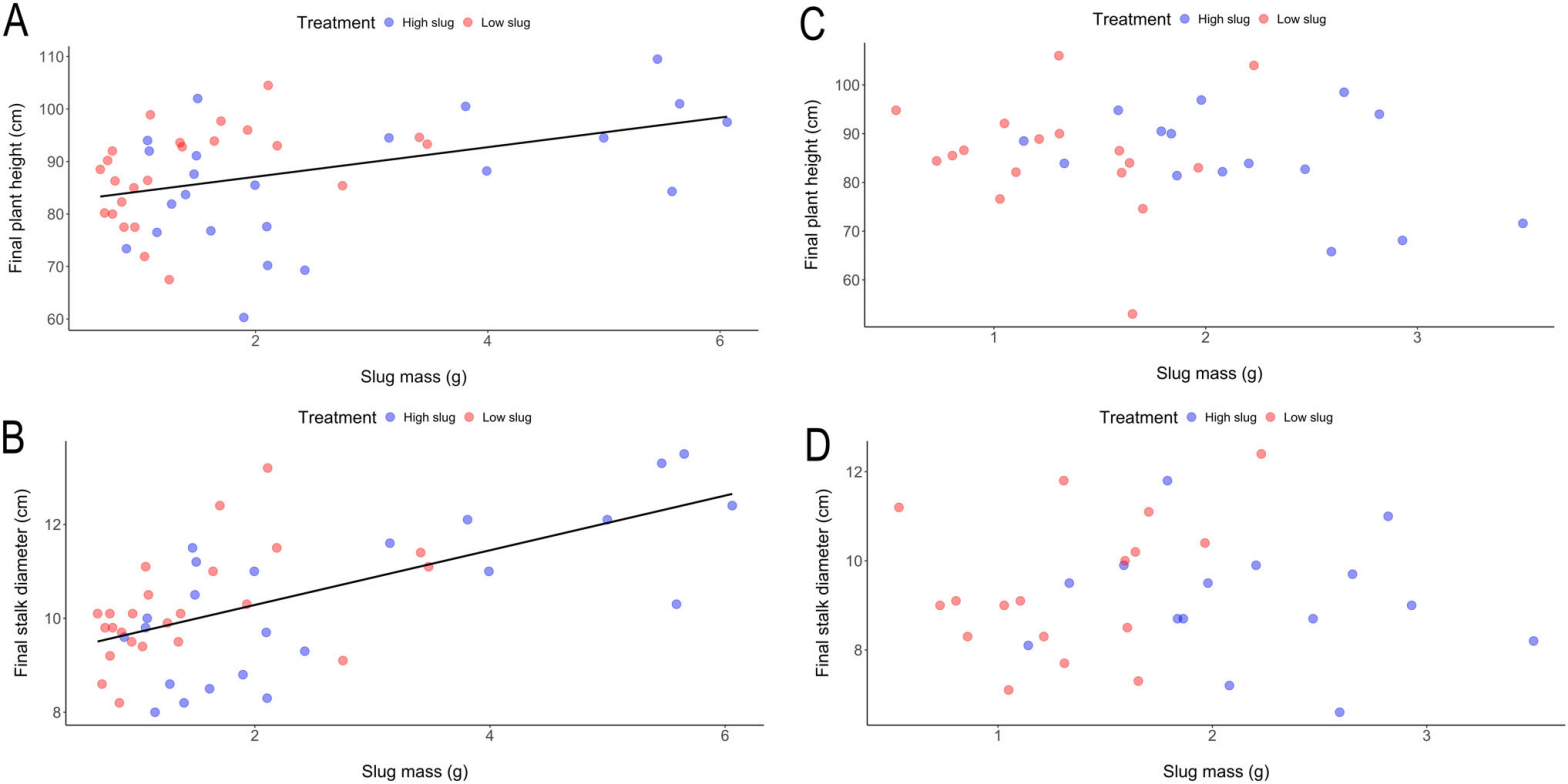
Regression analysis of plants subjected to slug damage at plant growth stage V1 (left‐hand column) and V3 (right‐hand column) showing the effect of the mass of slugs placed on plants on final A,C. plant height and B,D. stalk diameter. Best‐fit lines were generated using a regression model with a significance level at *P* < 0.05.

**Figure 6:**
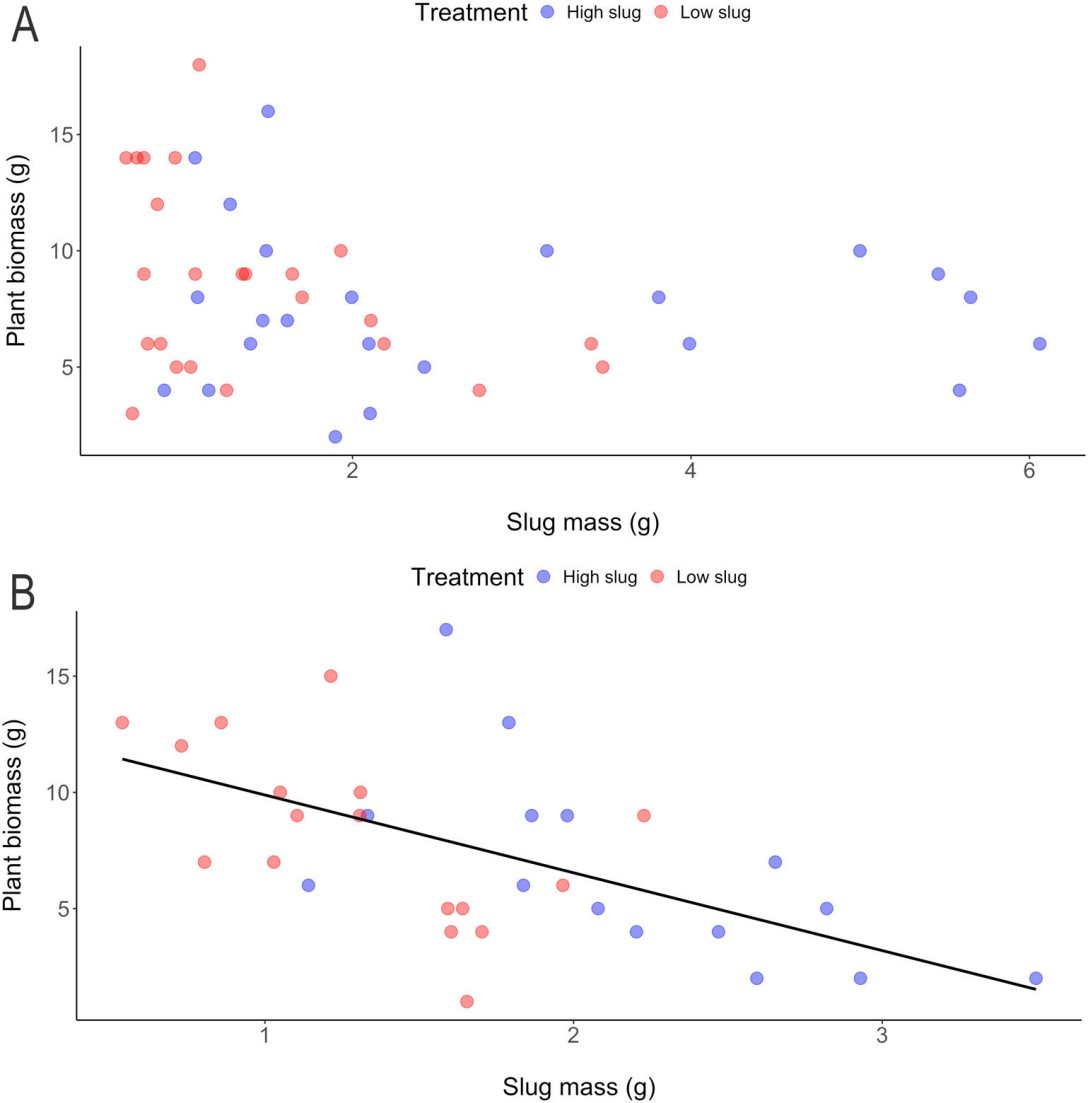
Regression analysis of plants subjected to slug damage showing the effect of the mass of slugs placed on plant biomass when plants were damaged at (A) V1 and (B) V3. Best‐fit line was generated using a regression model with a significance level at *P* < 0.05.

*Effects of slug mass on plant response*: For plants exposed to slugs at V1, the total mass of slugs placed on plants did not predict the leaf area remaining after they fed (*R* ^2^ = 0.005, *F*_1,48_ = 8.09, *P* = 0.6) nor did it predict plant biomass after 6 weeks of growth (*R* ^2^ = 0.05, *F*_1,45_ = 2.43, *P* = 0.13). Unexpectedly, we detected positive relationships between total mass of slugs placed on plants and final plant height (*R* ^2^ = 0.16, *F*_1,45_ = 8.38, *P* = 0.006) and stalk diameter (*R* ^2^ = 0.38, *F*_1,45_ = 27.8, *P* < 0.0001), such that height and diameter increased with increasing slug mass (Fig. 5). For plants damaged by slugs at growth stage V3, we did not detect similar patterns between mass of slugs and plant height or stem diameter (Fig. 5; plant height *vs* slug mass: *R* ^2^ = 0.05, *F*_1,30_ = 1.55, *P* = 0.22; final stalk diameter: *R* ^2^ = 0.00003, *F*_1,30_ = 0.0009, *P* = 0.98). Slug mass did, however, predict plant biomass after 6 weeks of growth such that plant biomass decreased as the mass of slugs on plants increased (Fig. 6: *R* ^2^ = 0.34, *F*_1,30_ = 15.6, *P* < 0.0005).

### 3.2 Assessment of slug damage to corn plants in field experiments

**Figure 7:**
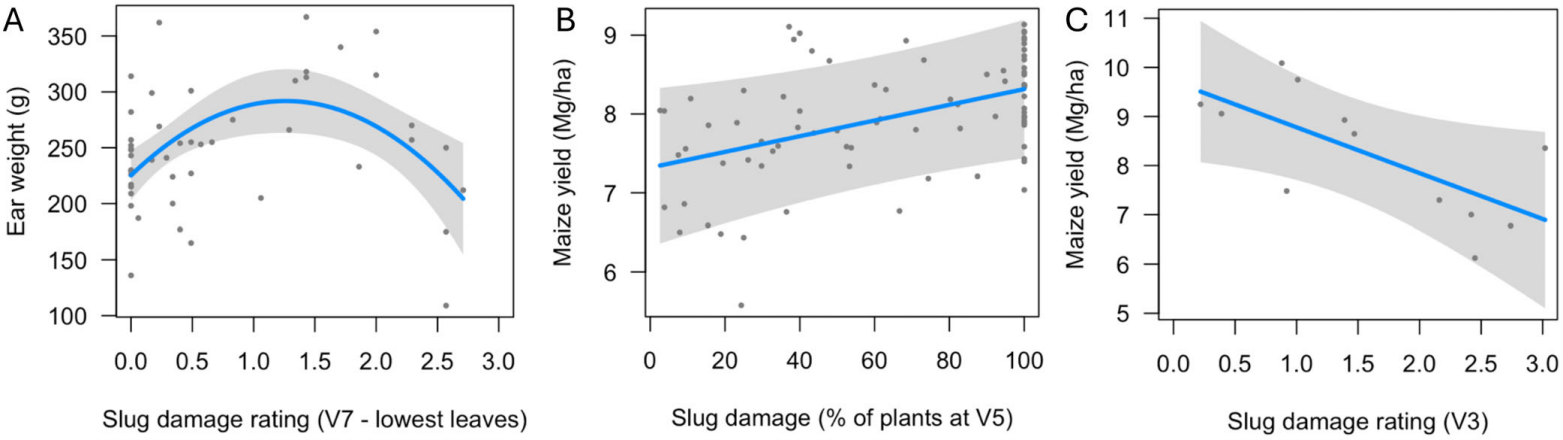
Partial regression plots for relationships for: (A) Slug damage (% defoliation of the lowest four leaves at V7) and ear weight of individual maize plants at harvest (2010; *n* = 48 plants, split among four plots). The shaded area shows the 95% confidence interval for the slope. (B) Maize yield and the percent of plants that received slug damage at growth stage V5. The data were gathered over 5 years in a cropping systems experiment (*n* = 16 split‐split plots nested in four plots per year). (C) Slug damage and maize yield in a 2012 field experiment testing the influence of seed treatments on maize and its invertebrate community (*n* = 12 plots; there was no significant effect of seed treatment on yield).

For our assessment in 2010, we found that the relationship between slug damage and ear weight was better described by a quadratic rather than a linear function (Table 1). Despite the overall significance of the regression (*F*_2,45_ = 6.10, *P* = 0.005), the function including slug damage and its square explained relatively little variation in ear weight of individual plants (*R* ^2^ = 0.18, Fig. 7(a)). Furthermore, the quadratic nature of the relationship indicated that ear weight increased with slug damage up to a damage rating of ~1.25 (corresponding to ~31% leaf area removed), then declined at greater levels of damage (Fig. 7(a)).

**Table 1:** Statistical results from a quadratic regression investigating the relationship between mean slug damage at V7 on the lowest four leaves, and ear weight (g) of mature corn ears. Individual corn plants (*n* = 48 plants, distributed among four plots) were followed across the growing season in 2010

Model term	Slope	SE	*t*	*P*
Slug damage rating (V7)	105	30	3.49	0.001
Slug damage rating (V7)^2^	−42	12	−3.38	0.001

Across 5 years in a cropping systems experiment, maize yield in a no‐till maize‐soy rotation was positively related to the percentage of seedlings that were damaged by slugs at V5 (Fig. 7(b)), an effect that was marginally significant (Table 2). The slope in the latter model suggested that a 10% increase in the proportion of seedlings damaged by slugs increased yield by 0.1 ± 0.05 mg/ha (~1% of total yield). Slug damage was distributed from zero to 100% of seedlings (mean percentage: 64 ± 3.9).

**Table 2:** Statistical results from a mixed effects model analyzing the relationship between the percent of seedlings damaged by slugs at V5 and maize yield, in a cropping systems field experiment over 5 years (*n* = 4 plots per year)

Model term	Slope	SE	df	*t*	*P*
Slug damage (% at V5)	0.01	0.005	17.7	1.85	0.08
Soil fertility treatment	−0.72	0.31	60.7	−2.34	0.02
Cutworm damage (% at V5)	−3.76	3.56	72.0	−1.06	0.29
Other caterpillar damage (% at V5)	−0.78	1.31	63.1	−0.60	0.55

Soil fertility treatment (chemical fertilizer [the baseline] or manure) and cutworm damage were included as covariates that may have influenced yield independently of slug damage. *T*‐tests used Kenward‐Roger's method to estimate degrees of freedom.

In contrast, in the seed treatment experiment from 2012 with a wider range of slug pressure, there was a significant negative effect of slug damage on maize yield (Table 3, Fig. 7c). An increase in slug damage by one point decreased yield by 0.93 ± 0.38 mg/ha (~11% of total yield). Slug damage was distributed from a damage rating of 0.2 (5% of leaf area removed) to 3 (75% of leaf area removed; mean rating: 1.59 ± 0.27). In this study, unlike the other, we also observed slugs feeding below‐ground on maize seedlings.

**Table 3:** Statistical results from a linear regression investigating the relationship between percent of seedlings damaged by slugs at V5 and maize yield in a single year (2012) with heavy slug damage in a field experiment (*n* = 12 plots)

Model term	Slope	SE	*t*	*P*
Slug damage rating (V3)	−0.93	0.38	−2.43	0.04
Seed treatment	0.44	0.63	0.70	0.50
Cutworm damage (% at V5)	−0.11	0.10	−1.09	0.31

Seed treatment (none [the baseline] or neonicotinoid) and cutworm damage were included as covariates that may have influenced yield independently of slug damage.

## 4 DISCUSSION

Previous applied research exploring the putative influence of slugs on plant productivity has not often included actual slugs^30^, ^31^ and usually has not compared effects of slugs with those imposed by mechanical damage.^34^, ^44^ Such studies have concluded that slug damage can reduce plant growth and yield^30^, ^31^, ^34^, ^45^ but have assumed that tissue removal accounts for most of the injury inflicted by slugs. A few studies with slugs or snails, however, and ample research with insects, indicate that cues associated with attacking animals can impose growth or reproductive costs on plants beyond wounding and tissue removal.^5^, ^6^, ^7^, ^8^, ^22^, ^23^

In our greenhouse experiments, we expected that slug feeding and mechanical wounding would both negatively influence plant growth, but that slug feeding would be more damaging for maize. Our results suggest for young maize plants (V1 to V3 growth stage) that slug and mechanical damage are similar (Figs 1, 2, 3, 4). Because our high and low slug‐damage treatments tended to remove more tissue from plants than their respective mechanical‐damage treatments (Fig. 1), our experimental design limits our capacity to conclude that slugs more strongly influenced plant growth than mechanical damage. After 6 weeks of growth, high‐slug damage had the strongest negative effect on plant response. Relative to undamaged plants, high‐slug damage significantly lowered leaf area remaining (Fig. 1, V1 and V3 plants), height (Fig. 2, V1 and V3 plants), stem diameter (Fig. 3, V3 plants), and plant biomass (Fig. 4, V1 and V3 plants) while the other treatments tended to be intermediate. Therefore, the treatment that removed the most tissue had the strongest influence on maize growth.

Notably, the high‐mechanical and low‐slug treatments removed similar amounts of tissue (Fig. 1(a), (b)); therefore, comparison of the two treatments can provide insight on whether slug damage can be more problematic for plant growth. In all our measurements over 6 weeks of growth following damage at V1 or V3, the two treatments were statistically similar (Figs 2, 3, 4), suggesting that slug damage is no worse for maize plants than mechanical damage. Moreover, despite removal of different amounts of tissue, statistical similarities across slug‐damaged and mechanical‐damaged treatments (*i.e*., plant height at V3 [Fig. 2(b)], stalk diameter at V1 and V3 [Fig. 3], and biomass at V1 and V3 [Fig. 4]) further suggest that slug and mechanical damage equally influenced plant performance.

Given the similarities we measured between plants damaged mechanically or by slugs, cues from saliva (and/or mucus) of slugs may not be detected by maize plants as insect saliva can be,^46^ or if slug cues are detected, they do not trigger defensive responses that are evident *via* simple measures of short‐term plant performance. Like our results, previous research comparing plant‐defense responses between slugs and caterpillars concluded that slugs may be stealthy feeders because they induced few if any responses from a variety of plant species.^44^ Further, slug‐derived cues might be expected to have little influence on maize plants because slugs are generalist feeders that feed opportunistically on plants, fungi, and even animal tissues (*e.g*., insect eggs, dead animals of various types).^9^ Slugs, therefore, may have not exerted enough selection pressure over evolutionary time on some plant species to recognize their cues as well as those from insect species, which can be more specialized on particular plant taxa. More specifically, while *D. reticulatum* can be a significant pest of maize,^10^ the two species have limited evolutionary history: *D. reticulatum* appears to have evolved in northern Europe^47^ whereas maize evolved in southern Mexico. Nevertheless, some plant species are sensitive to cues from terrestrial mollusks. For example, one slug species (*D. reticulatum*) and one snail species (*Cornu aspersum*), and cues from their locomotory mucus, triggered induced defenses in brassicaceous species (*e.g*., *Brassica*, *Arabidopsis*),^22^, ^23^ which is consistent with their evolutionary histories—western Asia and southern Europe are important centers of diversification for brassicaceous species^48^ whereas *D. reticulatum, C. aspersum*, and their relatives appear to be native to Europe, including parts of the Mediterranean region.^47^, ^49^

Regression analyses of our greenhouse results suggested that the direction of the effect of slugs depended on growth stages of maize plants. For V1‐stage plants, positive relationships of plant height or stalk diameter with slug mass indicate that greater amounts of slugs on plants induced growth that was still evident at the end of our 6‐week experiment (Fig. 5(a), (b)). Such induced plant growth could be interpreted as a form of tolerance called compensatory growth, which has been previously reported for maize plants following root herbivory by beetle larvae.^50^, ^51^ Compensatory growth has also been documented following slug feeding on *Brassica*,^52^ but we are not aware of previous reports of compensatory responses by maize to slugs. Notably, when damaged by slugs, V3‐stage plants did not produce similar compensatory growth (Fig. 5(c), (d)), but rather showed a more expected negative relationship between plant biomass and slug mass (Fig. 6(b)). Conventional wisdom holds that younger plants should have weaker abilities than older plants to tolerate feeding damage and/or produce compensatory growth.^4^, ^53^ Therefore, it is unexpected that slug damage to V1‐stage plants induced greater growth than plants damaged at V3‐stage. Wild relatives of maize strongly compensated for damage from a stem‐boring caterpillar species while a modern maize cultivar did not,^54^ so perhaps compensatory responses were disfavored by artificial selection for modern maize varieties or happened to be traits of the hybrids we used, as has been found previously.^27^ Nevertheless, our results indicate that when they are attacked at certain growth stages, some maize varieties can compensate for slug damage. It would be helpful to understand the genetic mechanisms underlying such responses to facilitate breeding for tolerance to slugs. Moreover, it would be useful to explore whether there is a cost to compensatory growth in the presence of other early‐season stressors (*e.g*. drought or competition).

From the longest of our three field experiments, unexpectedly, we found similar evidence that maize can tolerate and possibly compensate for early season slug damage. For data collected over 5 years (2011–2015), we found that maize yield was positively associated with amounts of damage that plants experienced by growth stages V5 (Fig. 7(a)). This field‐based research quantified yield and slug damage over several years and thus appears to be a more robust evaluation of responses of maize to slug feeding than our 6‐week‐long greenhouse experiment. Rather than just 48 h of slug exposure, depending on the year, maize plants received damage for 30–51 days (from emergence to V5), but most of this damage was mild (*i.e*., it was concentrated between 0.5 and 1.5 on a 0–4 rating scale^32^) even though most plants received damage (Fig. 7(a)). Nevertheless, an increase in the percentage of plants with slug damage from 10 to 90% resulted in a yield increase of 0.79 mg/ha (Fig. 7). A skeptical interpretation of these data could be that levels of slug damage were associated with other factors that positively influenced corn yield, but it is notable that these field‐based results are consistent with the positive influence of slugs from our greenhouse experiment.

In contrast, our experiment on seed treatments occurred in one year (2012) in a low‐lying field, revealing a more expected negative relationship between yield and slug damage. An increase in slug rating from 0.5 to 1.5 resulted in a yield reduction of 0.95 mg/ha. In 2012, plants appear to have experienced more severe slug damage (several plots averaging scores of 2 or higher; Fig. 7(b)). In addition to the aboveground slug damage, in this experiment in 2012 we also anecdotally observed belowground damage by slugs but not in the 5‐year study, which could help to explain its different outcome. Future research could clarify the significance of belowground feeding by slugs by quantifying this distinct type of injury. Overall, it appears, not surprisingly, that higher levels of slug damage are problematic for maize, though maize growth and recovery would depend heavily on abiotic conditions during the rest of the growing season.^34^

Remarkably, results of our assessment of the influence of slug damage from 2010 are consistent with both other field experiments and seem to integrate across them. The plants in the 2010 assessment grew from May through October and experienced a range of damage on young leaves from 0 to about 70% defoliation. At the end of the season, their productivity (as measured by ear mass) depended in part on the amount of slug damage that plants received; lower amounts of damage were associated with increasing ear masses whereas higher levels of slug damage were associated with decreasing ear masses (Fig. 7(c)). The positive association of ear mass (a proxy for yield) with slug damage may suggest that maize plants can compensate for lower levels of herbivory but not higher levels of damage, similar to the results we presented above. Encouragingly, a previous paper also found a quadratic response of maize plants to slug feeding that appears to suggest mild positive responses of maize yield to low levels of slug damage and negative responses to higher levels of slug damage.^34^

## 5 CONCLUSION

Our results suggest that slug damage to maize plants imposes costs similar to mechanical damage, but better controlled experiments are needed to address this question definitively including the potential influence of belowground feeding by slugs. Unexpectedly, in both our greenhouse and field experiments we found evidence that low amounts of slug damage can induce what appears to be compensatory growth in maize plants, but this induced growth likely depends on many factors, including the growth stage of plants, amounts of damage they receive, and subsequent abiotic conditions. Our results also reinforce that low levels of slug herbivory are unlikely to negatively influence crop yield and do not require management.^10^ Also, results from our field experiments confirmed that productivity of maize plants suffers with higher levels of slug feeding, which is not surprising and is consistent with slug management strategies known to growers—mainly that crops should germinate promptly and grow quickly to outgrow the risk from slugs in spring.^10^

With growing interest in no‐till farming, more fields are likely to build conditions that are conducive to slugs, so slugs are likely to become a more common problem in field‐crop production and it will be helpful to understand the costs of their damage. More research, therefore, is necessary on a wider variety of crop species to clarify the influence of slugs on plant growth. Moreover, with ongoing climate change, some regions, including the Mid‐Atlantic U.S., are expected to experience warmer winters and more precipitation, rendering no‐till crop production in these areas even more vulnerable to slug populations. It is therefore critical to develop understanding of slug ecology, including effects of their feeding, so research can have enough context to help develop effect slug management plans.^10^, ^55^

## FUNDING INFORMATION

Funding for the greenhouse portion of this research came from USDA NIFA award 2019–68012‐29818, which supported M.B. Funding for the field research present here came from many sources, including USDA's Northeast Sustainable Agriculture Research and Education program (GNE11‐014, LNE09‐291, LNE13‐129), the Pennsylvania Department of Agriculture (44123709), the Maryland Grain Producers Utilization Board, the College of Agricultural Sciences at Penn State, Penn State's Department of Entomology, and USDA's National Institute of Food and Agriculture and Hatch Appropriations under Projects #PEN04600, #PEN04606 and Accession #1009362.

## CONFLICT OF INTEREST

Authors have no conflicts of interest to declare.

## Data Availability

The data that support the findings of this study are openly available in ScholarSphere at https://doi.org/10.26207/r3te-wj74.
